# Smoking status and endothelial function in Japanese men

**DOI:** 10.1038/s41598-020-80012-x

**Published:** 2021-01-08

**Authors:** Haruki Hashimoto, Tatsuya Maruhashi, Takayuki Yamaji, Takahiro Harada, Yiming Han, Yuji Takaeko, Yasuki Kihara, Kazuaki Chayama, Chikara Goto, Yoshiki Aibara, Farina Mohamad Yusoff, Shinji Kishimoto, Masato Kajikawa, Ayumu Nakashima, Yukihito Higashi

**Affiliations:** 1 Department of Cardiovascular Medicine, Graduate School of Biomedical and Health Sciences, Hiroshima University, Hiroshima, Japan; 2 Department of Gastroenterology and Metabolism, Institute of Biomedical and Health Sciences, Graduate School of Biomedical and Health Sciences, Hiroshima University, Hiroshima, Japan; 3 Hiroshima International University, Hiroshima, Japan; 4 Department of Cardiovascular Regeneration and Medicine, Research Institute for Radiation Biology and Medicine (RIRBM), Hiroshima University, 1-2-3 Kasumi, Minami-ku, Hiroshima, 734-8551 Japan; 5 Department of Stem Cell Biology and Medicine, Hiroshima University Graduate School of Biomedical Sciences, Hiroshima, Japan; 6 Division of Regeneration and Medicine, Medical Center for Translational and Clinical Research, Hiroshima University Hospital, Hiroshima, Japan

**Keywords:** Cardiology, Risk factors

## Abstract

It is established that smoking is a major risk factor of atherosclerosis. Endothelial dysfunction occurs in the initial step in the pathogenesis of atherosclerosis and plays a critical role in the development of atherosclerosis. The purpose of this study was to evaluate the association between smoking status and endothelial function in detail in men. We measured flow-mediated vasodilation (FMD) in 2209 Japanese men including 1181 men who had never smoked and 1028 current smokers. All of the participants were divided into five groups by smoking pack-years: never smoker group (= 0), light smoker group (> 0 to 10), moderate smoker group (> 10 to 20), heavy smoker group (> 20 to 30) and excessive smoker group (> 30). FMD significantly decreased in relation to pack-years (6.6 ± 3.4% in the never smoker group, 6.8 ± 3.0% in the light smoker group, 6.5 ± 2.9% in the moderate smoker group, 5.9 ± 2.9% in the heavy smoker group, and 4.9 ± 2.7% in the excessive smoker group; *P* < 0.001). After adjustment for age (≥ 65 years), body mass index, systolic blood pressure, low-density lipoprotein cholesterol, glucose, and year of recruitment, FMD was significantly smaller in the excessive smoker group than in the never smoker group as a reference group (OR 1.95, 95% CI 1.42 to 2.67; *P* < 0.001). These findings suggest that FMD decreases with an increase in the number of cigarettes smoked and that excessive smoking is associated with endothelial dysfunction. Cigarette smoking is harmful to vascular function in men who are heavy smokers.

## Introduction

Cigarette smoking kills over five million people worldwide every year despite the fact that cigarette control policies have been implemented and the prevalence of smoking has declined in many countries^1^. It has been reported that approximately 10% of all the adult deaths from cardiovascular diseases are attributed to cigarette use^2^. Smoking per se is a major risk factor of atherosclerosis^3^. It is thought that smoking plays a critical role in the maintenance and development of atherosclerosis through an increase in low-density lipoprotein cholesterol levels, decrease in high-density lipoprotein cholesterol level, increase in catecholamine level, increase in the amount of fibrinogen, increase in reactive oxygen species (ROS), enhancement of platelet aggregation, increase in insulin resistance, and activation of Rho-associated kinase^4–6^.

Endothelial dysfunction occurs in the initial step in the pathogenesis of atherosclerosis and plays a critical role in the development of atherosclerosis^7,8^. Traditional cardiovascular risk factors are associated with endothelial dysfunction. It is thought that smoking also plays a critical role in the pathogenesis and development of atherosclerosis by, at least in part, endothelial dysfunction. Several investigations, including us, have clearly shown that smoking impairs endothelial function^6,9–11^. In addition, it has been shown that smoking is an independent predictor for endothelial dysfunction. However, the relationship between smoking status and endothelial function has not been shown in detail. How much does smoking affect endothelial function?

Recently, measurement of flow-mediated vasodilation (FMD) in the brachial artery has been widely used for assessing endothelial function in humans^12–15^. Measurement of FMD is useful for assessment of atherosclerosis from the early stage to end stage of atherosclerosis. It has been shown that FMD is an independent predictor of cardiovascular outcomes^16^.

In the present study, we evaluated the association of smoking status with endothelial function assessed by FMD in men.

## Results

### Relationship between smoking and endothelial function in current smokers in men

We evaluated the relationship between smoking and endothelial function in current smokers in men. The characteristics of 2209 subjects (1181 men who had never smoked and 1028 current smokers) are summarized in Table 1. The mean value of FMD in the 2209 subjects was 6.2 ± 3.2%. FMD was significantly smaller in current smokers than in men who had never smoked (5.9 ± 2.9% vs. 6.6 ± 3.4%, *P* < 0.001).

**Table 1:** Clinical characteristics of never and current smokers in men.

Variables	Total (n = 2209)	Never smoker (n = 1181)	Current smoker (n = 1028)	*P* value
Age, y	48 ± 14	47 ± 16	50 ± 12	< 0.001
Body mass index, kg/m^2^	23.4 ± 3.5	23.3 ± 3.5	23.6 ± 3.6	0.01
Systolic blood pressure, mmHg	128 ± 16	127 ± 15	129 ± 17	< 0.001
Diastolic blood pressure, mmHg	79 ± 12	77 ± 12	81 ± 12	< 0.001
Heart rate, bpm	65 ± 12	65 ± 12	65 ± 11	0.33
Total cholesterol, mmol/L	5.04 ± 0.88	5.04 ± 0.88	5.04 ± 0.88	0.82
Triglycerides, mmol/L	1.35 ± 0.71	1.24 ± 0.64	1.47 ± 0.76	< 0.001
HDL cholesterol, mmol/L	1.45 ± 0.39	1.53 ± 0.39	1.40 ± 0.36	< 0.001
LDL cholesterol, mmol/L	2.97 ± 0.80	2.97 ± 0.80	2.97 ± 0.83	0.86
Glucose, mmol/L	5.72 ± 1.44	5.66 ± 1.44	5.77 ± 1.44	< 0.001
HbA1c, %	5.7 ± 0.7	5.6 ± 0.8	5.7 ± .0.7	0.23
**Medical history, n (%)**				
Hypertension	973 (44.1)	487 (41.2)	486 (47.3)	0.005
Dyslipidemia	1059 (47.9)	510 (43.2)	549 (53.4)	< 0.001
Diabetes mellitus	262 (11.9)	120 (10.2)	142 (13.8)	0.008
Previous cardiovascular and cerebrovascular disease	250 (11.3)	131 (11.1)	119 (11.6)	0.74

All results are presented as mean ± SD.

HDL, high-density lipoprotein; LDL, low-density lipoprotein.

**Figure 1:**
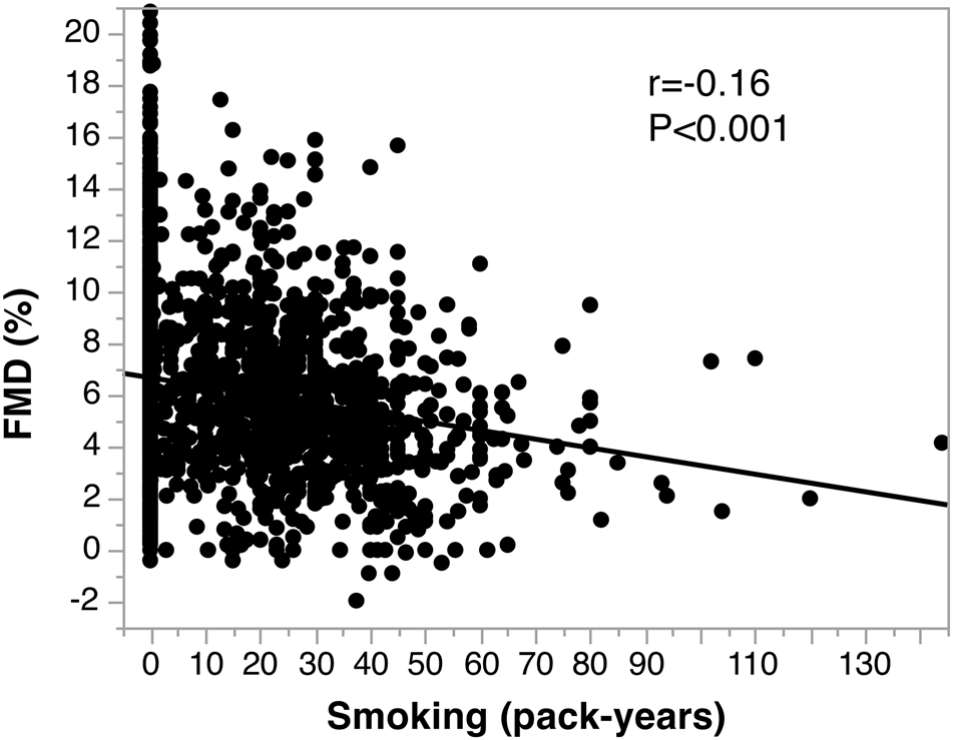
Scatter plots show the relationships between flow-mediated vasodilation (FMD) and smoking status in current smokers in men.

Univariate analysis revealed that pack-years was significantly correlated with FMD (r = -0.16, *P* < 0.001) (Table 2 and Fig. 1). After adjustment for age, body mass index (BMI), systolic blood pressure, low-density lipoprotein cholesterol, glucose, and year of recruitment, the association between pack-years and FMD (β = -0.07, *P* < 0.001) was significant (Table 3).

**Table 2:** Univariate analysis of the relation between FMD and variables in never smokers and current smokers of men.

Variables	r	95% CI		*P* value
Age, y	− 0.40	− 0.44	− 0.37	< 0.001
Body mass index, kg/m^2^	− 0.22	− 0.26	− 0.18	< 0.001
Systolic blood pressure, mmHg	− 0.21	− 0.25	− 0.17	< 0.001
Diastolic blood pressure, mmHg	− 0.14	− 0.18	− 0.09	< 0.001
Heart rate, bpm	− 0.05	− 0.09	− 0.01	0.03
Total cholesterol, mmol/L	0.04	0.00	0.09	0.04
Triglycerides, mmol/L	− 0.17	− 0.21	− 0.13	< 0.001
HDL cholesterol, mmol/L	0.11	0.07	0.15	< 0.001
LDL cholesterol, mmol/L	0.04	0.00	0.09	0.04
Glucose, mmol/L	− 0.29	− 0.32	− 0.25	< 0.001
HbA1c, %	− 0.16	− 0.20	− 0.12	< 0.001
Smoking, pack-years	− 0.16	− 0.20	− 0.12	< 0.001

HDL, indicates high-density lipoprotein; LDL, low-density lipoprotein; FMD, flow-mediated vasodilation; 95% CI 95% confidence interval.

**Table 3:** Multivariate analysis of the relations between FMD and variables in never smokers and current smokers in men.

Variables	β	*P* value
Intercept		< 0.001
Age, y	− 0.33	< 0.001
Body mass index, kg/m^2^	− 0.16	< 0.001
Systolic blood pressure, mmHg	− 0.11	< 0.001
LDL cholesterol, mmol/L	0.03	0.11
Glucose, mmol/L	− 0.05	0.01
Year of recruitment	0.22	< 0.001
Smoking, pack-years	− 0.07	< 0.001

Initial factors included in the model were age, body mass index, systolic blood pressure, LDL cholesterol, glucose, and year of recruitment.

FMD, flow-mediated vasodilation; LDL, low-density lipoprotein.

**Figure 2:**
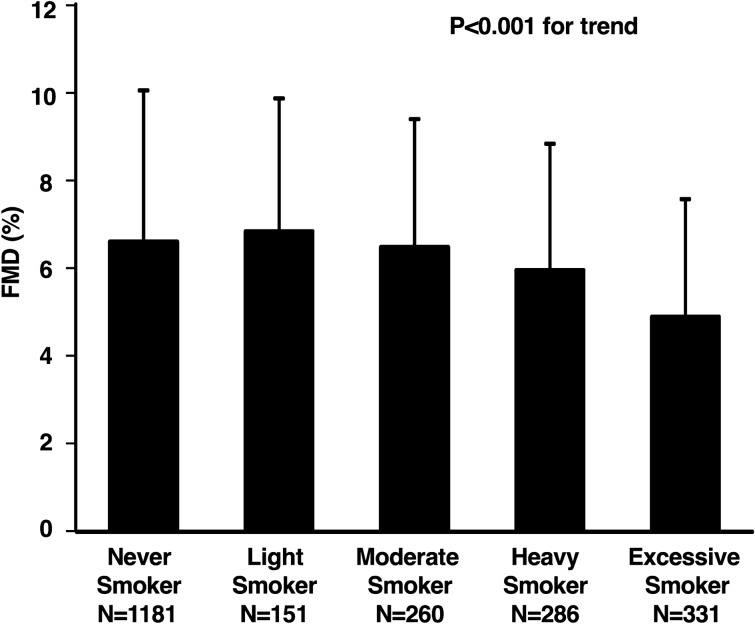
Bar graphs show flow-mediated vasodilation (FMD) in the never smoker group, the lights smoker group, the moderate smoker group, the heavy smoker group and the excessive smoker group in current smokers in men.

The clinical characteristics of men who had never smoked and current smokers categorized according to smoking pack-years are summarized in Table 4. Values of FMD were 6.6 ± 3.4% in the never smoker group, 6.8 ± 3.0% in the light smoker group, 6.5 ± 2.9% in the moderate smoker group, 5.9 ± 2.9% in the heavy smoker group, and 4.9 ± 2.7% in the excessive smoker group (Fig. 2). FMD was significantly smaller in the heavy and excessive smoker groups than in the never smoker group. FMD was significantly higher in the light smoker group than in the heavy and excessive smoker groups. We took the never smoker group as a reference and derived the OR for the low quartile of FMD (< 4.08%) in subjects who had never smoked and current smokers in men. After adjustment for age (≥ 65 years), BMI, systolic blood pressure, low-density lipoprotein cholesterol, glucose, and year of recruitment, FMD was significantly smaller in the excessive smoker group than in the reference group: light (OR: 1.57, 95% CI: 0.99 to 2.51; *P* = 0.06), moderate (OR: 1.01, 95% CI: 0.68 to 1.50; *P* = 0.97), heavy (OR: 1.38, 95% CI: 0.97 to 1.96; *P* = 0.08), and excessive (OR: 1.95, 95% CI: 1.42 to 2.67; *P* < 0.001) (Table 5).

**Table 4:** Clinical characteristics of never smokers and current smokers in men.

Variables	Total (n = 2209)	Never smoker pack year = 0 (n = 1181)	Light smoker 0 < pack year≦10 (n = 151)	Moderate smoker 10 < pack year≦20 (n = 260)	Heavy smoker 20 < pack year≦30 (n = 286)	Excessive smoker 30 < pack year (n = 331)	*P* value
Age, y	48 ± 14	47 ± 16	39 ± 12	45 ± 10	51 ± 8	58 ± 9	< 0.001
Body mass index, kg/m^2^	23.4 ± 3.5	23.3 ± 3.5	23.1 ± 3.2	23.5 ± 3.3	23.9 ± 3.6	23.7 ± 3.9	0.01
Systolic blood pressure, mmHg	128 ± 16	127 ± 15	127 ± 16	127 ± 17	130 ± 17	132 ± 19	< 0.001
Diastolic blood pressure, mmHg	79 ± 12	77 ± 12	78 ± 13	80 ± 12	83 ± 11	82 ± 12	< 0.001
Heart rate, bpm	65 ± 12	65 ± 12	64 ± 12	64 ± 10	64 ± 10	67 ± 12	0.03
Total cholesterol, mmol/L	5.04 ± 0.88	5.04 ± 0.88	5.02 ± 0.88	5.07 ± 0.91	5.09 ± 0.83	4.97 ± 0.85	0.32
Triglycerides, mmol/L	1.35 ± 0.71	1.24 ± 0.64	1.30 ± 0.63	1.40 ± 0.73	1.54 ± 0.85	1.54 ± 0.75	< 0.001
HDL cholesterol, mmol/L	1.45 ± 0.39	1.53 ± 0.39	1.45 ± 0.34	1.40 ± 0.36	1.40 ± 0.36	1.40 ± 0.41	< 0.001
LDL cholesterol, mmol/L	2.97 ± 0.80	2.97 ± 0.80	2.97 ± 0.83	3.05 ± 0.80	3.00 ± 0.80	2.90 ± 0.80	0.23
Glucose, mmol/L	5.72 ± 1.44	5.66 ± 1.44	5.44 ± 1.33	5.50 ± 1.00	5.66 ± 1.05	6.22 ± 1.94	< 0.001
HbA1c, %	5.7 ± 0.7	5.6 ± .0.8	5.5 ± 0.6	5.6 ± .0.6	5.7 ± .0.6	5.9 ± .0.8	< 0.001
**Medical history, n (%)**							
Hypertension	973 (44.1)	487 (41.2)	47 (31.1)	89 (34.2)	143 (50.0)	207 (62.5)	< 0.001
Dyslipidemia	1059 (47.9)	510 (43.2)	64 (42.4)	125 (48.1)	163 (57.0)	197 (59.5)	< 0.001
Diabetes mellitus	262 (11.9)	120 (10.2)	9 (6.0)	18 (6.9)	38 (13.3)	77 (23.3)	< 0.001
Previous cardiovascular and cerebrovascular disease	250 (11.3)	131 (11.1)	7 (4.6)	17 (6.5)	31 (10.8)	64 (19.3)	< 0.001

All results are presented as mean ± SD.

HDL, high-density lipoprotein; LDL, low-density lipoprotein.

**Table 5:** Odds ratios and 95% confidence intervals for low quartile of FMD according to pack-years in never smokers and current smokers in men.

Pack-years	Unadjusted OR (95% CI) *P* value	Adjusted* OR (95% CI) *P* value
Never	1 (reference)	1 (reference)
Light 0 < Pack-years≦10	0.90 (0.59–1.36) 0.61	1.57 (0.99–2.51) 0.06
Moderate 10 < Pack-years≦20	0.72 (0.51–1.02) 0.06	1.01 (0.68–1.50) 0.97
Heavy 20 < Pack-years≦30	1.10 (0.82–1.49) 0.52	1.38 (0.97–1.96) 0.08
Excessive 30 < Pack-years	2.19 (1.69–2.83) < 0.001	1.95 (1.42–2.67) < 0.001

FMD, flow-mediated vasodilation.

Low quartile of FMD indicates less than 4.08%.

*Adjusted also for age (≥ 65 years), body mass index, systolic blood pressure, low-density lipoprotein cholesterol, glucose, and year of recruitment.

## Discussion

In the present study, we demonstrated that FMD was significantly smaller in smokers than in subjects who had never smoked, that FMD decreased with an increase in smoking pack-years and that FMD was significantly decreased in the light, moderate, heavy and excessive smoker groups compared with that in subjects who had never smoked. After adjustment for cardiovascular risk factors, FMD was significantly smaller in the excessive smoker group than in the never smoker group in men.

Our results support the results of previous studies showing that smoking impairs endothelial function^9–11,17–20^. Smoking, as well as other cardiovascular risk factors, is an independent predictor of endothelial function^16^. In the present study, we investigated the relationship between smoking status and endothelial function in detail. It is well known that smoking and cardiovascular risk factors are relevant confounding factors for each other. Indeed, in the present study, after adjustment of cardiovascular risk factors, light, moderate, and heavy smoking were not associated with endothelial dysfunction, while endothelial function was impaired in relation to an increase in smoking pack-years. However, we emphasize that excessive smoking per se is associated with endothelial dysfunction even after adjustment of cardiovascular risk factors.

In the present study, FMD value in excessive smokers was reduced by approximately 1% compared with that in never smokers. Meta-analyses revealed that a reduction in the FMD value of 1% was associated with an approximately 13% increase in the odds of cardiovascular events independent of traditional cardiovascular risk factors^21,22^. Indeed, in the present study, the prevalence of previous cardiovascular events was higher in excessive smokers that in never smokers. A study is needed to confirm future cardiovascular events in excessive smokers compared with those in never smokers.

Although the mechanisms by which smoking impairs endothelial function are complex and multifactorial, several mechanisms for smoking-induced endothelial dysfunction have been postulated. Smoke from tobacco has various types and large amounts of reactive oxygen species^23,24^. Murohara et al. reported that contents of cigarette smoke caused vasoconstriction in the porcine coronary artery through superoxide-induced degradation of nitric oxide (NO)^25^. In smokers, circulating levels of the antioxidant ascorbic acid are decreased and oxidative stress markers such as oxidative low-density lipoprotein, F2-isoprostanes and 8-hydroxy-2′-deoxyguanosine are increased^26,27^. Even a single bout of smoking decreases circulating levels of nitrite and nitrate and antioxidants such as ascorbic acid, cysteine, methionine, and uric acid^28^ Administration of ascorbic acid restored the impairment of endothelium-dependent vasodilation in smokers^29^. It has been shown that xanthine oxidase, one of the enzymatic sources of ROS, is activated in heavy smokers and that production of ROS induced by xanthine oxidase contributes to the endothelial dysfunction in those subjects^30^. Higman et al. reported that endothelium-dependent vasodilation in the saphenous vein of smokers was impaired through a deficiency of the endothelial NO synthase (eNOS) coenzyme tetrahydrobiopterin, while the concentrations of eNOS in endothelial cells of the saphenous vein were similar in smokers and subjects who had never smoked^31^. It is thought that a deficiency of tetrahydrobiopterin predominately results in the production of ROS rather than NO in smokers. These findings suggest that both activation of oxidative stress and attenuation of the antioxidant system co-exist, leading to a decrease in NO bioavailability and resulting in endothelial dysfunction in smokers. In addition, we previously showed that vascular response to fasudil, a Rho-associated kinase inhibitor, is significantly greater in smokers than in non-smokers, indicating that the activity of Rho-associated kinase is enhanced in smokers^6,10^. It has been shown that an increase in Rho-associated kinase activity mediated a decrease in NO bioavailability through inhibition of eNOS mRNA stability and eNOS protein phosphorylation at Ser 1177 via the Akt/PI3K pathway, leading to a vicious circle between activation of Rho-associated kinase and inactivation of the eNOS/NO pathway^32^.

Our study has a number of limitations. First, smoking status may be an inaccurate assessment since smoking status was calculated using questionnaires. We cannot deny the possibility that smoking status was underestimated or overestimated. However, a previous study showed that self-reports are accurate in most studies^33^. Second, we evaluated the relationship between smoking status estimated as absolute smoking pack-years and FMD. However, we did not evaluate the influence of differences in kinds of tobacco and nicotine content on FMD. Third, it has been shown that smoking cessation improves endothelial function^34^. The duration of smoking cessation was unclear in our database. After evaluation of the relationship between smoking status and endothelial function in a general population including former smokers, we selected current smokers who had not stopped smoking as study subjects. Fourth, several investigators have shown that circulating estrogen levels affect endothelial function in premenopausal women^35,36^. Unfortunately, we did not measure estrogen levels and did not ask about the menstrual cycle. Therefore, we did not evaluate the relationship between smoking status and endothelial function in women in the present study. Evaluation of the relationship between smoking status and FMD only in women after adjustment of the menstrual cycle would enable more specific conclusions concerning the role of smoking in endothelial function in women to be drawn. Fifth, the recruitment period is relatively long in the Hiroshima University Hospital registry (n = 574). In the present study, we confirmed that FMD was significantly smaller in the excessive smoker group than in the reference group after adjustment for year of recruitment. However, we cannot deny the possibility that the characteristics of the study participants are heterogenous depending on the time of recruitment and that differences in the characteristics of subjects have bias to results. Finally, in this study, 653 (29.6%) of the subjects were receiving anti-hypertensive drugs, 355 (16.1%) of the subjects were receiving lipid-lowering drugs and 186 (8.4%) of the subjects were receiving diabetic drugs. This study was performed without withholding medications since the subjects were recruited from health-screening examinations. It would have been inappropriate to withhold medications. However, Gokce et al. demonstrated that administration of vasoactive medication not including nitrates does not significantly influence the values of FMD and nitroglycerine-induced vasodilation^37^. In the present study, subjects who were receiving nitrates were excluded.

In conclusion, FMD decreases with an increase in the number of cigarettes smoked. Excessive smoking is associated with endothelial dysfunction. Cigarette smoking is harmful to vascular function in men who are heavy smokers.

## Methods

### Study subjects

A total of 6150 Japanese adults (4161 subjects from the Flow-mediated Dilatation Japan (FMD-J) Registry between June 2010 and May 2012 and 1989 subjects who underwent a health checkup at Hiroshima University Hospital between August 2007 and January 2019) were enrolled in this study. Subjects with unclear images of the brachial artery interfaces (n = 226) and subjects with missing information on smoking status (n = 886) were excluded. Several investigators have shown that circulating estrogen levels affect endothelial function in premenopausal women^35,36^. We did not measure estrogen levels and did not ask about the menstrual cycle. Therefore, women were excluded (n = 1387). It has been shown that smoking cessation improves endothelial function^34^. Because the duration of smoking cessation was unclear in our database, former smokers were excluded (n = 1442). Subjects who took anti-hypertensive drugs, lipid-lowering drugs or anti-diabetic drugs on the day of FMD measurement were also excluded since some drugs influence FMD*.* Finally, 2209 subjects were enrolled in this study. Detailed information on the subjects and protocol of the FMD-J study is publicity available^38^.

Hypertension was defined as systolic blood pressure of more than 140 mm Hg and/or diastolic blood pressure of more than 90 mm Hg, in a sitting position, measured on at least three different occasions. Diabetes was defined according to the American Diabetes Association or a previous diagnosis of diabetes^39^. Dyslipidemia was defined according to the third report of the National Cholesterol Education Program^40^.

All participants were divided into five groups by smoking pack-years: never smoker group (= 0), light smoker group (> 0 to 10), moderate smoker group (> 10 to 20), heavy smoker group (> 20 to 30) and excessive smoker group (> 30).

Hiroshima University ethical committee approved the study protocol. The study was executed in accordance with the Good Clinical Practice guidelines. Informed consent for participation in the study was obtained from all subjects. The protocol was registered in the University Hospital Medical Information Network Clinical Trials Registry (UMIN000003759).

### Study protocol

A total of 2209 adults answered a questionnaire about smoking status. We measured vascular responses to reactive hyperemia in the brachial artery in all subjects^41^. Additional details are available in the online-only Data Supplement.

### Measurement of FMD

EMD was measured by using UNEXEF18G (UNEX Co, Nagoya, Japan) as previously described^41^. Additional details are available in the online-only Data Supplement.

### Statistical analysis

Results are presented as means ± SD for continuous variables and as percentages for categorical variables. Statistical significance was set at a level of *P* < 0.05. Continuous variables were compared by using one-way analysis of variance (ANOVA) for multiple groups. Categorical variables were compared by means of the χ2 test. The relations between FMD and smoking status was determined by Spearman’s correlation. Multiple logistic regression analysis was performed to identify independent variables associated with a lower quartile of FMD (< 4.08). According to the American Heart Association statement on criteria for evaluation of novel markers of cardiovascular risk, a new biomarker should be tested for significance only after all established risk factors have already been included in the model^42^. Hypertension, dyslipidemia, diabetes, aging, smoking, and obesity are commonly known cardiovascular risk factors. These cardiovascular risk factors are independent contributing factors of vascular function and structure. Also, because the recruitment period is long, we cannot deny the possibility that the characteristics of the study participants are heterogenous depending on the time of recruitment and that differences in the characteristics of subjects have bias to results. Indeed, we focused on the measurement of FMD in relatively young subjects during the first half period from 2007 to 2013 and measured FMD in subjects of all ages during the second half period from 2014 to 2019. Thus, we adjusted age (≥ 65 years), BMI, systolic blood pressure, low-density lipoprotein cholesterol, glucose and year of recruitment (during the first half period from 2007 to 2013 and during the second half period from 2014 to 2019) for multiple logistic regression analysis. The data were processed using the software package Stata, version 9 (Stata Co, College Station, TX).

## Supplementary Information

Supplementary Information.
