# Supplementary material for: Smoking status and endothelial function in Japanese men
Source: Sci Rep. 2021 Jan 8;11:95. doi: 10.1038/s41598-020-80012-x (PMC7794366; doi:10.1038/s41598-020-80012-x)
Supplement: Supplementary file 1 — Supplementary Information. [file 41598_2020_80012_MOESM1_ESM.docx]

**Online Supplemental Data**

**Smoking Status and Endothelial Function in Japanese Men**

Brief title: Smoking and endothelial function

Haruki Hashimoto, MD;^1^ Tatsuya Maruhashi, MD, PhD;^1^ Takayuki Yamaji, MD;^1^ Takahiro Harada, MD;^1^ Yiming Han, MD;^1^ Yuji Takaeko, MD;^1^ Yasuki Kihara, MD, PhD;^1^ Kazuaki Chayama, MD, PhD;^2^ Chikara Goto, PhD;^3^ Yoshiki Aibara, MS;^4^ Farina Mohamad Yusoff;^4^ Shinji Kishimoto, MD, PhD;^4^ Masato Kajikawa, MD;^5^ Ayumu Nakashima, MD, PhD;^5^

Yukihito Higashi, MD, PhD, FAHA^4,6^

^1^Department of Cardiovascular Medicine, Graduate School of Biomedical and Health Sciences, Hiroshima University, Hiroshima, Japan

^2^Department of Gastroenterology and Metabolism, Institute of Biomedical and Health Sciences, Graduate School of Biomedical and Health Sciences, Hiroshima University Hiroshima, Japan

^3^Hiroshima International University, Hiroshima, Japan

^4^Department of Cardiovascular Regeneration and Medicine, Research Institute for Radiation Biology and Medicine, Hiroshima University, Hiroshima, Japan

^5^Department of Stem Cell Biology and Medicine, Hiroshima University Graduate School of Biomedical Sciences, Hiroshima, Japan

^6^Division of Regeneration and Medicine, Medical Center for Translational and Clinical Research, Hiroshima University Hospital, Hiroshima, Japan

Address for correspondence: Yukihito Higashi, MD, PhD, FAHA

Department of Cardiovascular Regeneration and Medicine,

Research Institute for Radiation Biology and Medicine (RIRBM), Hiroshima University

1-2-3 Kasumi, Minami-ku, Hiroshima 734-8551, Japan

Phone: +81-82-257-5831 Fax: +81-82-257-5831

E-mail: [yhigashi@hiroshima-u.ac.jp](mailto:yhigashi@hiroshima-u.ac.jp)

**Methods**

**Study protocol**

The subjects fasted the previous night for at least 12 hours and they were instructed to abstain from alcohol, smoking and caffeine at least 12 hours prior to the measurements. The study began at 8:30 AM. The subjects were kept in the supine position in a quiet, dark, air-conditioned room (constant temperature of 22°C-25°C) throughout the study. A 23-gauge polyethylene catheter was inserted into the left deep antecubital vein to obtain blood samples. Thirty minutes after maintaining the prone position, FMD were measured. The observers were blind to the form of examination.

**Measurement of FMD**

Vascular response to reactive hyperemia in the brachial artery was used for assessment of endothelium-dependent FMD.^1,2^ A high-resolution linear artery transducer was coupled to computer-assisted analysis software (UNEXEF18G, UNEX Co, Nagoya, Japan) that used an automated edge detection system for measurement of brachial artery diameter. A blood pressure cuff was placed around the forearm. The brachial artery was scanned longitudinally 5-10 cm above the elbow. When the clearest B-mode image of the anterior and posterior intimal interfaces between the lumen and vessel wall was obtained, the transducer was held at the same point throughout the scan by a special probe holder (UNEX Co) to ensure consistency of the image. Depth and gain setting were set to optimize the images of the arterial lumen wall interface. When the tracking gate was placed on the intima, the artery diameter was automatically tracked, and the waveform of diameter changes over the cardiac cycle was displayed in real time using the FMD mode of the tracking system. This allowed the ultrasound images to be optimized at the start of the scan and the transducer position to be adjusted immediately for optimal tracking performance throughout the scan. Pulsed Doppler flow was assessed at baseline and during peak hyperemic flow, which was confirmed to occur within 15 sec after cuff deflation. Blood flow velocity was calculated from the color Doppler data and was displayed as a waveform in real time. The baseline longitudinal image of the artery was acquired for 30 sec, and then the blood pressure cuff was inflated to 50 mm Hg above systolic pressure for 5 min. The longitudinal image of the artery was recorded continuously until 5 min after cuff deflation. Pulsed Doppler velocity signals were obtained for 20 sec at baseline and for 10 sec immediately after cuff deflation. Changes in brachial artery diameter were immediately expressed as percentage change relative to the vessel diameter before cuff inflation. FMD was automatically calculated as the percentage change in peak vessel diameter from the baseline value. Percentage of FMD [(Peak diameter - Baseline diameter)/Baseline diameter] was used for analysis. Blood flow volume was calculated by multiplying the Doppler flow velocity (corrected for the angle) by heart rate and vessel cross-sectional area (-r2). Reactive hyperemia was calculated as the maximum percentage increase in flow after cuff deflation compared with baseline flow.

**References**

1. Maruhashi, T., et al. Nitroglycerine-induced Vasodilation for Assessment of Vascular Function: A Comparison with Flow-mediated Vasodilation. *Arterioscler Thromb Vasc Biol* **33** 1401-1408 (2013).
2. Takaeko, Y., et al. Association of extremely high levels of high-density lipoprotein cholesterol with endothelial dysfunction in men. *J Clin Lipidol* **13** 664-672 (2019).

**Acknowledgments**

The authors would like to thank all patients who participated in this study. In addition, we thank Miki Kumiji, Megumi Wakisaka, Ki-ichiro Kawano and Satoko Michiyama for their excellent secretarial assistance; FMD-J investigators of Takayuki Hidaka, MD, PhD; Shuji Nakamura, MD, PhD; Junko Soga, MD, PhD; Yuichi Fujii, MD, PhD; Naomi Idei, MD; Noritaka Fujimura, MD, PhD; Shinsuke Mikami, MD, PhD; Yumiko Iwamoto, MD; Akimichi Iwamoto, MD, PhD; Takeshi Matsumoto, MD, PhD; Nozomu Oda, MD, PhD (Department of Cardiovascular Medicine, Hiroshima University Graduate School of Biomedical Sciences, Hiroshima, Japan); Kana Kanai, PhD; Hraruka Morimoto, PhD (Department of Cardiovascular Regeneration and Medicine, Research Institute for Radiation Biology and Medicine, Hiroshima University, Hiroshima, Japan); Tomohisa Sakashita, MD, PhD; Yoshiki Kudo, MD, PhD (Department of Obstetrics and Gynecology, Hiroshima University Graduate School of Biomedical Sciences, Hiroshima, Japan); Taijiro Sueda, MD, PhD (Department of Surgery, Hiroshima University Graduate School of Biomedical Sciences, Hiroshima, Japan); Hirofumi Tomiyama, MD, PhD, FAHA; Akira Yamashina, MD, PhD (Department of Cardiology, Tokyo Medical University, Tokyo, Japan); Bonpei Takase, MD, PhD, FAHA (Division of Biomedical Engineering, National Defense Medical College Research Institute, Tokorozawa, Japan); Takahide Kohro, MD, PhD (Department of Cardiology, Tokyo Medical University, Tokyo, Japan); Toru Suzuki, MD, PhD (Cardiovascular Medicine, University of Leicester, Leicester, UK); Tomoko Ishizu, MD, PhD (Cardiovascular Division, Institute of Clinical Medicine, University of Tsukuba, Ibaraki, Japan); Shinichiro Ueda, MD, PhD (Department of Clinical Pharmacology and Therapeutics, University of the Ryukyu School of Medicine, Okinawa, Japan); Tsutomu Yamazaki, MD, PhD (Clinical Research Support Center, Faculty of Medicine, The University of Tokyo, Tokyo, Japan); Tomoo Furumoto, MD, PhD (Department of Cardiovascular Medicine, Hokkaido University Graduate School of Medicine, Hokkaido, Japan); Kazuomi Kario, MD, PhD (Division of Cardiovascular Medicine, Jichi Medical University School of Medicine, Tochigi, Japan); Teruo Inoue, MD, PhD (Department of Cardiovascular Medicine, Dokkyo Medical University, Mibu, Tochigi, Japan); Shinji Koba, MD, PhD (Department of Medicine, Division of Cardiology, Showa University School of Medicine, Tokyo, Japan); Kentaro Watanabe, MD, PhD (Department of Neurology, Hematology, Metaboism, Endocrinology and Diabetology, Yamagata University School of Medicine, Yamagata, Japan); Yasuhiko Takemoto, MD, PhD (Department of Internal Medicine and Cardiology, Osaka City University Graduate School of Medicine, Osaka, Japan); Takuzo Hano, MD, PhD (Department of Medical Education and Population-based Medicine, Postgraduate School of Medicine, Wakayama Medical University, Wakayama, Japan); Masataka Sata, MD, PhD (Department of Cardiovascular Medicine, Institute of Health Biosciences, The University of Tokushima Graduate School, Tokushima, Japan); Yutaka Ishibashi, MD, PhD (Department of General Medicine, Shimane University Faculty of Medicine, Izumo, Japan); Koichi Node, MD, PhD (Department of Cardiovascular and Renal Medicine, Saga University, Saga, Japan); Koji Maemura, MD, PhD (Department of Cardiovascular Medicine, Nagasaki University Graduate School of Biomedical Sciences, Nagasaki, Japan); Yusuke Ohya, MD, PhD (The Third Department of Internal Medicine, University of the Ryukyus, Okinawa, Japan); Taiji Furukawa, MD, PhD (Department of Internal Medicine, Teikyo University School of Medicine, Tokyo, Japan); Hiroshi Ito, MD, PhD (Department of Cardiovascular Medicine, Okayama University Graduate School of Medicine, Dentistry and Pharmaceutical Sciences, Japan); Hisao Ikeda, MD, PhD (Faculty of Fukuoka Medical Technology, Teikyo University, Omuta, Japan).
